# Biological rhythms and epilepsy treatment

**DOI:** 10.3389/fneur.2023.1153975

**Published:** 2023-08-11

**Authors:** Jon Andreas Rugstad Næsgaard, Leif Gjerstad, Kjell Heuser, Erik Taubøll

**Affiliations:** ^1^Faculty of Medicine, Institute of Clinical Medicine, University of Oslo, Oslo, Norway; ^2^Department of Neurology, Division of Clinical Neuroscience, ERGO – Epilepsy Research Group of Oslo, Oslo University Hospital, Oslo, Norway

**Keywords:** chronotherapy, biological rhythms, personalized medicine, chronopharmacology, epilepsy treatment, seizure patterns

## Abstract

Approximately one-third of patients with epilepsy are drug-refractory, necessitating novel treatment approaches. Chronopharmacology, which adjusts pharmacological treatment to physiological variations in seizure susceptibility and drug responsiveness, offers a promising strategy to enhance efficacy and tolerance. This narrative review provides an overview of the biological foundations for rhythms in seizure activity, clinical implications of seizure patterns through case reports, and the potential of chronopharmacological strategies to improve treatment. Biological rhythms, including circadian and infradian rhythms, play an important role in epilepsy. Understanding seizure patterns may help individualize treatment decisions and optimize therapeutic outcomes. Altering drug concentrations based on seizure risk periods, adjusting administration times, and exploring hormone therapy are potential strategies. Large-scale randomized controlled trials are needed to evaluate the efficacy and safety of differential and intermittent treatment approaches. By tailoring treatment to individual seizure patterns and pharmacological properties, chronopharmacology offers a personalized approach to improve outcomes in patients with epilepsy.

## Introduction

With up to one-third of patients with epilepsy defined as drug-refractory (1), resistance to conventional treatment with antiseizure medication ASM contributes to considerable morbidity, calling for novel treatment approaches. Adjusting pharmacological treatment to physiological variations in seizure susceptibility, and drug responsiveness (chronopharmacology), is a promising strategy to increase efficacy and tolerance.

The phenomenon of rhythmic seizure occurrence has been recognized for centuries (2). A circadian seizure pattern is frequently seen in patients with juvenile myoclonic epilepsy (JME) (3), while longer rhythms, like catamenial epilepsy, are found in about one-third of women with epilepsy who are of child-bearing age (4). With new technology for recording, seizure patterns are identified in an increasing number of patients. For instance, Spencer et al. concluded that all 134 patients monitored using intracranial EEG had patterns in their epileptiform activity (5).

Given the rhythmicity of epilepsy and a considerable treatment gap, this narrative review aims to provide a general overview of basic biological foundations for rhythms in seizure activity, present cases illustrating clinical implications of seizure patterns, and briefly discuss how chronopharmacological strategies may improve treatment.

## Mechanisms of biological rhythms

Biological rhythms are essential to most physiological processes and are commonly categorized as either circadian (about 24 h), ultradian (shorter than 24 h), or infradian (longer than 24 h) (6). This section will discuss circadian and infradian rhythms as these are most important in human epilepsy.

### Circadian rhythms

A thorough description of the circadian clock system is beyond the scope of this article, and readers are referred to Ayyar et al. (7) for a comprehensive review. In short, the circadian rhythm is based on self-sustaining oscillations in cells throughout the body, regulated by the suprachiasmatic nucleus (SCN) in the hypothalamus. The activity of the SCN is synchronized with the environment through input, primarily visual stimulus from specific retinal cells. The SCN relays this rhythm to the rest of the organism through the autonomic nervous system and endocrinological processes (particularly variations in melatonin and cortisol secretion) (8).

### Infradian rhythms

The most important infradian rhythm in epileptology is the menstrual cycle and the periods with the highest risk of seizure are perimenstrual (C1), preovulatory (C2), and luteal in anovulatory cycles (C3) (9). The common feature in these situations is an increased estrogen-to-progesterone ratio (10). Several studies have reinforced the hypothesis that estrogen has a proconvulsant effect, whereas progesterone has anticonvulsant properties (9, 11). D’amore et al. also found that interictal spikes (IIS) were closely linked to the four-day menstrual cycle in rats, and that these IIS rhythms disappeared in individuals that were ovariectomized (12). On a general note, catamenial epilepsy demonstrates how endocrine factors may alter excitability and thereby contribute to patterns in seizure occurrence.

Apart from catamenial epilepsy, information about the effect of chronotherapeutic treatment in patients with infradian seizure rhythms is lacking. Nevertheless, there is reason to expect benefits from adjusting treatment to seizure risks. Most importantly, this would imply high ASM concentration in periods of high risk and fewer side effects in low-risk periods. Additionally, it could modulate the regulation of receptors and downstream mediators, possibly enhancing the long-term efficacy of ASM.

Regarding infradian rhythms, one must account for the contributions of external factors to the periodic occurrence of seizures, possibly mimicking a biological rhythm (13). For instance, alcohol consumption or delayed bedtimes during weekends may increase seizure rates (14). Similar effects could manifest yearly around holidays.

Another plausible explanation of infradian rhythms is that different biological rhythms interact to form interference patterns. In a model where the seizure threshold is constant and rhythms with distinct periods alter neuronal excitability, one would expect constructive interference, adding up the effects of the various meters to create longer-term seizure patterns (15).

## Rhythmicity of epilepsy – case reports and clinical studies

There is substantial evidence suggesting that seizure patterns correlate with specific clinical features. Amengual-Gual et al. (16) have summarized typical high-risk periods for different epileptic foci and semiology types. Among their most important findings is that seizures with frontal, parietal, or multilobar origin occur mostly during sleep, whereas those with occipital or temporal origin typically occur in wakefulness. The authors also found a peak period of the highest seizure incidence for some foci (16). For instance, occipital lobe epilepsy occurs most frequently between 6 a.m. and 9 a.m. Regarding semiology, tonic seizures are most frequent during sleep, particularly right after midnight, whereas clonic seizures primarily happen during the daytime. Patterns in seizure occurrence are relevant in multiple clinical contexts, illustrated by the four following case reports.

### Circadian rhythms

#### Patient 1: wake-up seizures

The patient is a 42-year-old woman with epilepsy diagnosed after a generalized tonic–clonic seizure (GTCS) that occurred early in the morning at age 14. When asked, she reported both absences and myoclonic jerks for some time, misinterpreted by her parents as inattention and clumsiness. EEG showed generalized epileptogenic activity, and she was diagnosed with JME. To treat her condition, valproate has been used in various doses from the time of diagnosis, while lamotrigine was started five years later. Levetiracetam and perampanel were briefly tried but discontinued due to seizure worsening and side effects, respectively. Recently, topiramate was added, leading to one year of seizure freedom from GTC seizures, but seizures have now returned. Both the GTCSs and the myoclonic jerks predominantly occurs in the morning, exacerbated by stress and sleep deprivation. Due to this strict circadian seizure pattern, she regained her driver’s license.

**Comment**: This pattern is characteristic of JME. Pedersen and Petersen reported that 53.7% of patients had GCTSs primarily on awakening, and 62.8% had most myoclonic seizures on awakening (17). Moreover, sleep deprivation is a typical risk factor (3). Seizures related to awakening are also characteristic of West syndrome (18). Overall, mornings and evenings are reported to be the periods of highest seizure frequency in patients with circadian rhythms (13). Some have argued that this may be due to low drug concentrations at these times, but, this does not explain why specific epilepsy syndromes show a higher seizure incidence at certain times. However, different epilepsy syndromes are usually treated with specific ASMs. The different drugs may be affected differently by circadian rhythms, accounting for why particular syndromes show highest seizure frequencies at different times. Evidence suggests that administering drugs to maintain elevated plasma concentrations during periods of heightened seizure risk could be advantageous for treating patients with well-defined circadian rhythms (19). Therefore, it would be reasonable to anticipate positive outcomes from administering the medication in the evening for this patient.

#### Patient 2: seizure pattern related to sleep

The patient is a 20-year-old male with epilepsy from age 5 due to polymicrogyria in the right frontoparietal region. Except for one GTCS shortly after the time of diagnosis, he exclusively had focal seizures with impaired awareness (FIAs) and FA (focal aware) seizures during the night. There were usually between two and five seizures per night several times a week. The episodes lasted between 30 s and a minute, starting with a snoring sound. He then got up on his elbows and sometimes had lip-smacking and swallowing movements. His EEGs repeatedly showed spike-sharp wave activity in the right centrofrontal region, worsening during sleep. Levetiracetam initially improved sleep quality but caused daytime restlessness and was discontinued. Oxcarbazepine and eslicarbazepine had little effect on seizures. Sulthiam, started from age 13, reduced EEG epileptic activity at night but had an uncertain effect on clinical seizures. Valproate was added but provided no additional benefit, however, there were problems with adherence, resulting in subtherapeutic serum ASM concentrations. Despite trying several ASMs, the patient still has seizures, and the circadian rhythmicity is preserved. The patient is now treated with a combination of valproic acid and sulthiam, which is administered in the morning and evening. Because of the robust circadian rhythm in seizures, he is permitted to hold a driving license.

**Comment**: Karoly et al. (13) found that about 80 percent of 1,118 people with epilepsy (PWE) had circadian seizure patterns and that 45 percent of all seizures occurred at nighttime. It has also been shown that seizures originating from frontal or parietal foci are more likely to occur during sleep (16). Sejal et al. explain that NREM sleep leads to synchronization of neuronal networks, probably increasing the likelihood of interictal epileptiform discharges (IEDs) (18). Similarly, seizures seldom occur in the less synchronized activity found in REM sleep (18). Like the prior case, this patient might have benefitted from ASM being administered only in the evenings. The presented case illustrates how strong seizure patterns are relevant for treatment decisions and for patients’ ability to live with epilepsy.

### Infradian rhythms

#### Patient 3: catamenial epilepsy

**Figure 1:**
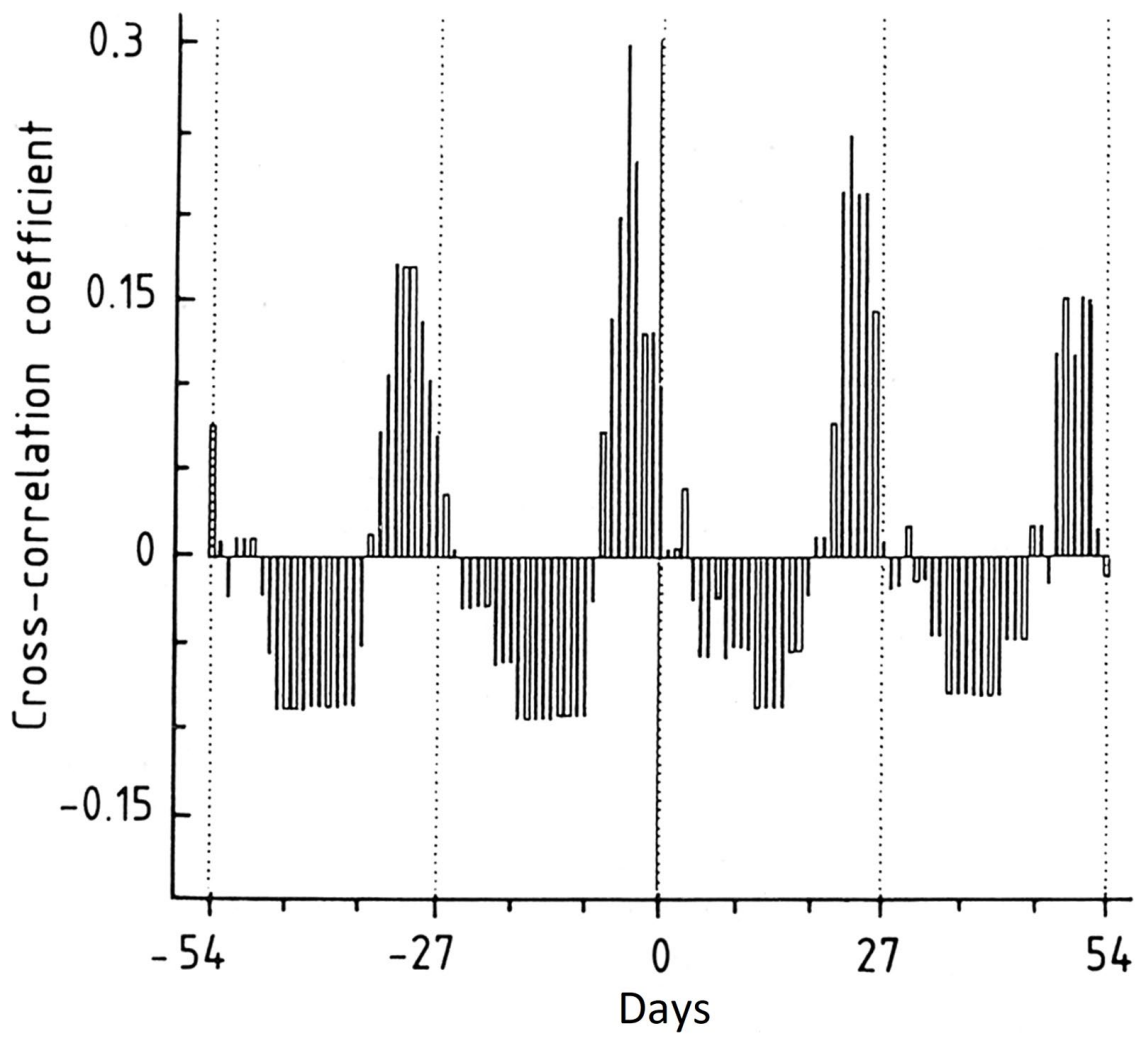
Cross-correlogram illustrating the relationship between the menstrual cycle and seizure occurrence in patient 3. The cross-correlation coefficient will range from −1 to +1. A cross-correlation coefficient of +1 means that the sets are identical with a complete overlap, i.e., 100% likelihood of seizures on that exact day. Day 0 is defined as the first day in the menstrual cycle. Note the monthly peaks, indicating a robust correlation between seizures and menstruation [Reproduced with permission from Taubøll et al. (20)].

The patient is a 55-year-old woman with her first seizure at age ten. She primarily had FIAs, but a few episodes generalized, and her condition stabilized with two to six seizures per month. A catamenial pattern was detected in her late teens when she was monitored for 323 days as part of a study investigating the temporal distribution of seizures (20). Approximately 90% of her seizures occurred during the premenstrual and menstrual phases, with a higher frequency premenstrually [Figure 1, modified from Taubøll et al. (21)]. The pattern continued for several years. During this period, she tried seven different ASMs without effect on seizure frequency. Additionally, 1,000 mg/day, acetazolamide was attempted from day 20 to day 3 of the cycle (day 1 defined as the first day of menstruation). This had a modest effect but was discontinued due to side effects. Cyclic dosing of clobazam was unsuccessful, and intermittent progesterone treatment was impracticable due to irregular menstrual cycles. Medroxyprogesterone acetate (MPA) was tried for over a year at a dose that stopped menstrual bleeding, resulting in a less predictable seizure pattern, causing the patient additional discomfort. Her seizure frequency declined in the last three years following menopause.

**Comment**: This patient presents a typical C1 seizure distribution, with most seizures in the perimenstrual period. Herzog et al. reported that 42.3% of all women with epilepsy had some catamenial rhythmicity. Of those, 35.7% had predominantly perimenstrual seizures (4). The patient also illustrates how irregular menstrual cycles complicate intermittent progesterone treatment. Furthermore, the case report illustrates that some patients prefer the predictability associated with regular seizures to the unpredictability of random attacks.

#### Patient 4: seizures related to thyroid hormones

The patient is a 28-year-old, otherwise healthy male with FIA seizures of unknown etiology, starting from age 14. During follow up, he also experienced two GTCS. Focal epileptogenic activity was found in the right temporal lobe, but MRI was normal. For four years, the patient’s seizures were remarkably regular. They were clustered in 2-4-day periods with up to 10 episodes per day followed by 5–6 weeks of seizure-free intervals. Seizure-precipitating factors were studied in detail, revealing a relationship between changes in thyroid hormone concentrations and seizure frequency. During the study, he was treated with carbamazepine 1,000 mg per day. The thyroxine (T4) levels exhibited a statistically significant variation within the reference range. Specifically, during seizure periods, defined as one day before to one day after a seizure event, the median T4 level measured 81 nmol/L. In contrast, between seizures, defined as periods exceeding five days from a seizure occurrence, the median T4 level was 72 nmol/L.

**Comment**: It is recognized that thyroid hormones may affect brain excitability, and seizures have been reported as the presenting symptom in 9 % of all admissions for thyrotoxicosis (22). In animals, both thyroxine and triiodothyronine lower the seizure threshold when exposed to electroshocks, while thyroidectomy has the opposite effect (23). Interventions aimed at reducing T4 levels could theoretically decrease seizure activity, but there are no interventional studies to support this theory. In addition, several thyroid hormone metabolites may have both pro- and anticonvulsive effects, but their clinical implications are still unknown (24). It is worth considering the close interaction between antiseizure medications (ASMs) like carbamazepine and thyroid hormones (25). However, the observed rhythmicity in our patient cannot be easily explained by changes in interactions or metabolites, as medication was kept unchanged throughout the study. Moreover, Taubøll et al. discuss the possibility of reverse causality or confounding variables to explain the rhythmic patterns in hormone concentrations (26).

## Treatment of epilepsy with a rhythmic seizure occurrence

The presented cases exemplify rhythmic seizure occurrences with diverse etiologies, demanding carefully adapted treatment approaches. The catamenial and thyroid hormone-dependent patterns may be managed by hormone therapy, whereas intermittent ASM treatment may counteract other infradian rhythms. The predictability of the presented patients’ seizure patterns illustrates the importance of biological rhythms for seizure prediction, both from an epidemiological and individual perspective. Studying patients with exceptional seizure patterns may increase our understanding of the mechanisms driving rhythmicity in epilepsy.

Seizure patterns may also help personalize treatment. Careful documentation of seizure occurrence, possibly supplemented by long-term neurophysiological monitoring, could help adjust treatment. Although the latter poses challenges, Duun-Henriksen et al. (27) demonstrated that long-term monitoring was possible using subcutaneous scalp electrodes. However, acquiring statistically processable data would require a certain number of seizures. In patients with infrequent seizures, one could instead use interictal epileptiform discharges as an indicator of epileptic activity. Karoly et al. found that spontaneous interictal spikes increased in periods of high seizure risk indicating that these could be used to assess the risk of seizure occurrence (28).

Given the apparent link between clinical features and patterns of seizure activity, one might use similar treatment schedules for patients with common etiology or semiology (16). Tailoring treatment to individuals or small groups is a type of personalized medicine that might help reduce over- and undertreatment. Another possible strategy is to target the fundamental rhythmical biological alterations that lead to increased seizure susceptibility. For instance, hormonal contraceptives alter the natural fluctuations of sex steroid hormones. Similarly, thyroidectomies protect model animals from seizures induced by electroshock (23, 29). Unfortunately, there is insufficient evidence to support targeting endocrinological factors influencing seizure risk in a clinical context, apart from sex steroid hormones.

### Chronopharmacological treatment

*Chronopharmacology* applies knowledge about biological rhythms to optimize pharmacological treatment strategies (30) and has been the most promising chronotherapeutic approach in epilepsy treatment. In principle, it can be applied for infradian and circadian rhythms but has mainly been utilized to optimize the treatment for patients with circadian seizure patterns.

When optimizing pharmacological treatment, one must also consider that neuronal excitability may be influenced by fluctuating drug responsiveness (31). The following paragraphs will introduce two phenomena influencing this: rhythmic variations in pharmacokinetics (chronokinetics) and fluctuations in pharmacodynamic properties (chronesthesy).

*Chronokinetics* can be divided into rhythmic variations in drug absorption, distribution, metabolism, and excretion. Regarding absorption, gastric pH, emptying rate and concentration of transporters in the gut endothelium vary with circadian periods (7). In addition, plasma protein binding of several ASMs fluctuates (32, 33), hepatic activity varies, and there is considerable change in renal clearance throughout the day (34). All these factors affect ASM concentration and must be considered to optimize drug efficacy. Translational studies have shown significant circadian variations in carbamazepine plasma concentration (35). There is also evidence of fluctuating ASM concentration in the cerebral extracellular space, but the interindividual differences are substantial (36).

*Chronesthesy* is mainly determined by the free fraction of the drug in plasma, target availability, and the number of key mediators in signaling pathways (7). For example, Kusunose et al. found circadian variation in the α-2δ-1 subunit of a calcium channel that is the target of gabapentin. This variation corresponded with the maximum binding capacity of gabapentin (37) and may therefore explain variations in drug efficacy. One way to study chronesthesy is to keep the drug concentration in plasma constant, counteracting chronokinetics, and measuring changing drug responsiveness. These conditions are challenging to maintain, and studies exploring chronesthesy in ASM are lacking.

## Discussion

As many people with epilepsy continue having seizures despite optimized conventional ASM treatment, there is a demand for new approaches. Chronopharmacology could offer personalized treatment tailored to individual variations in seizure activity, pharmacokinetics, and pharmacodynamics. Similar therapy is being established in various clinical contexts, like cancer treatment (38), rheumatology (39), and antihypertensive treatment (40).

The studies of chronotherapy in epilepsy are based on the principle of differential dosing, that is tailoring the drug concentration to the patient’s seizure risk. The largest trial was conducted by Yegnanarayan et al. (19), who included patients with subtherapeutic serum concentrations of ASM and randomized them to either change the time of administration or to increase the dose. Delaying the administration time outperformed dose elevation in reaching therapeutic serum concentrations and reducing seizure frequency. In another group, they found that a change of administration time to the evening reduced adverse effects significantly (19).

Guilhoto et al. (41) also significantly reduced seizure frequency when the fraction of ASM administered in the evening increased. Whereas Thome-Souza et al. (42) reported a reduction in seizures when patients were prescribed adjuvant clobazam with most of the dose in the evening. Sánchez Fernández et al. (43) found that in a case series with 29 patients having continuous spikes and waves while sleeping, 20 experienced at least a 25% reduction in spike–wave percentage when treated with diazepam orally before going to bed.

The studies evaluating the effects of differential dosing are heterogeneous regarding population and intervention, making it difficult to compare the results directly. They generally have a short follow-up, retrospective design (except for Yegnanarayan and Herzog), and few participants. Additionally, Guilhoto et al. (41) recruited from a tertiary center without a control group, thereby limiting the applicability to the larger population of people with epilepsy. Nonetheless, these trials show promising results and should be followed by extensive and carefully designed studies.

Some have also proposed using melatonin to decrease seizure activity. A Cochrane Review published in 2016 (44) concluded that there was too little evidence to conclude regarding the efficacy of melatonin as an add-on treatment in epilepsy. However, Verma et al. recently published the results of their RCT, displaying a considerable benefit of melatonin combined with valproic acid for adults with generalized epilepsy (45). These results are in accordance with the anticonvulsive effects of melatonin suggested by some translational studies (46–48).

Regarding catamenial epilepsy, cyclic hormonal treatment with progesterone metabolites is the most promising alternative (9, 49). In 2012, Herzog et al. (10) completed a double-blind RCT studying the effect of adjuvant progesterone therapy in women with epilepsy. They found a correlation between the fraction of perimenstrual seizures and the effect of progesterone as an add-on treatment in the luteal phase. There is also evidence to suggest that group stratification may be helpful, given apparent differences in pathophysiology between the C1, C2, and C3 groups. In addition to hormonal treatment, several different treatment options have been suggested for catamenial epilepsy, including intermittent cyclic treatments with benzodiazepines, increasing the dose of antiseizure drugs already in use, cycle suppressive treatment, and acetazolamide (4).

Given the burden of unpredictable seizures, Karoly et al. argue that seizure-predicting devices could increase the quality of life in epilepsy patients and enable targeted interventions. Using a dataset from self-reported episodes and a risk-calculating algorithm, they concluded that patients spent only 10.5% of their time in a high-risk state. However, they experienced 69% of their seizures during these short periods. Prospective studies have concluded with long-term decreased seizure risk and improved reported quality of life in patients treated with responsive neurostimulation (RNS) (50).

Finally, chronotherapy may not be limited to traditional ASM. Examining the circadian variations in the transcriptome could identify new targets for chronopharmacological interventions. There is extensive ongoing research to identify substances modulating core clock genes (CCGs) and their products (51). Considering the possible link between disturbances in CCG expression and seizure activity (52, 53), this endeavor may yield results.

## Conclusion

To summarize, treatment adjusted both to variations in seizure risk and pharmacological properties shows promise as a strategy to achieve better efficacy and tolerance of ASM. Both translational research and clinical trials have illustrated that chronopharmacology is feasible and showed beneficial effects. However, it is necessary to identify which patients should be offered chronotherapy and determine the optimal protocol for administering ASM.

Considering the formerly discussed limitations of the chronotherapy trials, there is a need for large-scale randomized controlled trials to evaluate the efficacy and safety of differential and intermittent treatment. As there are many reasons for seizure patterns, some interventions may be helpful only in specific subgroups, and further studies may identify these.

## Author contributions

JN wrote most of the manuscript. ET wrote sections of the manuscript and provided Figure 1. KH and LG made substantial contributions to the writing of several sections. All authors contributed to the article and approved the submitted version.

## Conflict of interest

The authors declare that the research was conducted in the absence of any commercial or financial relationships that could be construed as a potential conflict of interest.

## Publisher’s note

All claims expressed in this article are solely those of the authors and do not necessarily represent those of their affiliated organizations, or those of the publisher, the editors and the reviewers. Any product that may be evaluated in this article, or claim that may be made by its manufacturer, is not guaranteed or endorsed by the publisher.
